# Effects of Ischemia on the Migratory Capacity of Microglia Along Collagen Microcontact Prints on Organotypic Mouse Cortex Brain Slices

**DOI:** 10.3389/fncel.2022.858802

**Published:** 2022-06-16

**Authors:** Katharina Steiner, Christian Humpel

**Affiliations:** Laboratory of Psychiatry and Experimental Alzheimer’s Research, Department of Psychiatry and Psychotherapy, Medical University of Innsbruck, Innsbruck, Austria

**Keywords:** microcontact printing, ischemia, organotypic brain slices, brain-on-a-chip, oxygen-glucose deprivation

## Abstract

Ischemic stroke is a severe insult in the brain causing cell death, inflammation, and activation of microglia. Microglia are the immune cells of the brain and play a role in any inflammatory process during neurodegeneration. Microglia are round ameboid and migrate to the lesion site, where they differentiate into ramified forms and activated phagocytic microglia. On the other hand, microglia can also release growth factors to repair degeneration. The aim of the present study is to explore the migratory capacity of microglia after ischemic insults. Organotypic brain slices of the mouse cortex (300 μm) were prepared. In order to study migration, the slices were connected to collagen-loaded microcontact prints (with or without monocyte chemoattractant protein-1, MCP-1) on the membranes. Slices were stimulated with lipopolysaccharide (LPS) for maximal microglial activation. Ischemic insults were simulated with oxygen-glucose deprivation (OGD) and acidosis (pH 6.5) for 3 days. After 3 weeks in culture, slices were fixed and immunohistochemically stained for the microglial markers Iba1, CD11b and macrophage-like antigen. Our data show that Iba1+ microglia migrated along the microcontact prints, differentiate and phagocyte 1.0 μm fluorescent microbeads. LPS significantly enhanced the number of round ameboid migrating microglia, while OGD and acidosis enhanced the number of ramified activated microglia. The effect was not visible on slices without any μCP and was most potent in μCP with MCP-1. We conclude that OGD and acidosis activate ramification and exhibit a similar mechanism, while LPS only activates round ameboid microglia. Collagen-loaded microcontact prints connected to mouse brain slices are a potent method to study activation and migration of microglia *ex vivo*.

## Introduction

Microglia are the resident immune cells in the brain and are able to sense pathological stimuli or inflammatory signals, to produce inflammatory signals themselves, and to phagocytose infectious pathogens or injurious self-proteins (Wright-Jin and Gutmann, 2019). Alterations of the functional phenotype are accompanied by dynamic changes of the cell morphology. In the healthy mature CNS, microglia are morphologically characterized by a small soma and ramified processes (Savage et al., 2019). Microglia constantly scan their environment for exogenous or endogenous signals and shift their activities from sensing toward protecting, which is accompanied by marked morphological changes including the formation of a macrophage (Orihuela et al., 2016). Ameboid microglia are actively moving to sites of infectivity and phagocytose infectious pathogens or mutated proteins following chemotactic gradients. Furthermore, microglia increase their local densities by proliferation, rearrange surface molecules, change intracellular enzymes, and release proinflammatory cytokines or neurotrophic factors (Kettenmann et al., 2010).

The mechanisms by which microglia participate in neurodegenerative diseases is a double-edged sword (Hickman et al., 2018). Upon activation microglia migrate to a site of infectious and injurious agents following chemotactic gradients (Carbonell et al., 2005). Ion channels and transporters control microglial migration *via* inducing the retraction and shrinkage at the rear part of a migrating cell, while at the front part actin projection (lamellipodium) is extended (Schwab, 2001). Upon encountering extracellular aggregated proteins (e.g., beta amyloid in Alzheimer’s disease) or cell debris, microglia acquire either the proinflammatory M1 phenotype or the neuroprotective M2 phenotype. As soon as there is any defect in clearing due to protein aggregate overload or dysfunctional microglia, microglia become chronically proinflammatory causing the severe neurotoxicity and neurodegeneration. Microglial survival and maintenance and migration is highly dependent on cytokines, such as example monocyte-chemoattractant protein-1 (MCP-1 or CCL2) (Deng et al., 2009).

Organotypic brain slice cultures bridge the gap between *in vitro* cell cultures and *in vivo* animal experiments. They offer a good alternative system to *in vivo* animal studies and simultaneously permit to markedly reduce the number of animal experiments as multiple slices can be obtained from one brain. The key benefit of organotypic brain slice cultures is that the complex 3-dimensional architecture of the brain is preserved, simulating *in vivo* situations when cells are studied in their natural microenvironment. Brain regions of interest are cultured on a semipermeable membrane interface between a humidified atmosphere and the culture medium. The brain slices attach to the membrane and receive nutrition from the culture medium through semipermeable membrane. Meanwhile, the membrane interface technique is broadly used by several research groups. Microglia have been well studied in such organotypic brain slices for many years (Coltman and Ide, 1996; Czapiga and Colton, 1999; Mitrasinovic et al., 2005; Delbridge et al., 2020), including our lab where we have shown that GM-CSF can stimulate the proliferation and the migration of microglia from brain slices (Schermer and Humpel, 2002).

The ability to pattern proteins and other biomolecules onto substrates is important for capturing the spatial complexity of the extracellular environment. μCP emerged as a simple, effective and cheap method for patterning a wide range of biomolecules onto a variety of different background materials. This high-resolution (sub-μm range) patterning technique provides a potent tool to engineer diverse cells *via* microprinting proteins of interest in defined stripes. As μCP of single biomolecules onto semipermeable membranes was not successful in our hands, we developed and optimized a method where we can μCP proteins embedded in collagen type I (Steiner and Humpel, 2021). Collagen is the most abundant extracellular matrix protein and it provides a biocompatible, biodegradable, non-toxic, and versatile possibility to mimic both, the structural and biological properties. We have recently shown that we can μCP nearly any protein/peptide on semipermeable membranes and couple them to brain slices (Steiner and Humpel, 2021). Using this method, we microprinted the nerve growth factor (NGF) and could show selective growth of cholinergic neurons along these microprinted lanes when coupled to a brain slice containing cholinergic neurons (Steiner and Humpel, 2021).

There is clear evidence that microglia get activated after ischemic stroke; a PubMed search (January 2022) finds 215 original papers. Several nice reviews highlight the role of microglia in cerebral ischemia (Lees, 1993; Lai and Todd, 2006; Benakis et al., 2015; Gülke et al., 2018; Surinkaew et al., 2018; Gaire, 2021), but a detailed summary is out of focus in this present study. There is one important study by Skibo et al. (2000), who studied the effects of hypoxia on microglia in hippocampal slice cultures, especially on ultrastructure and lipocortin-1. They (Skibo et al., 2000) have shown that microglia in organotypic brain slices preserve their characteristic features and properties comparable to microglial forms *in vivo* and after experimental toxic hypoxia rapid changes in microglial ultrastructure were seen. Thus, we are confident that in the present study we can explore the migratory role of microglia in our organotypic brain slices, which may resemble an *in vivo* situation.

The aim of the present study was to investigate the effects of ischemia on microglial migration in a mouse organotypic brain slice model. In order to induce migration, we microcontact print the protein MCP-1 onto membranes and couple them to brain slices. Maximal microglial activation is induced by lipopolysaccharide (LPS). Ischemia is simulated with glucose-oxygen deprivation (OGD) or acidosis (pH 6.5) for 3 days. Our data show that LPS activated round ameboid microglia to migrate, while OGD and acidosis exhibited a similar mechanism but induced mainly ramification.

## Materials and Methods

### Organotypic Brain Slices of the Mouse Cortex

**FIGURE 1:**
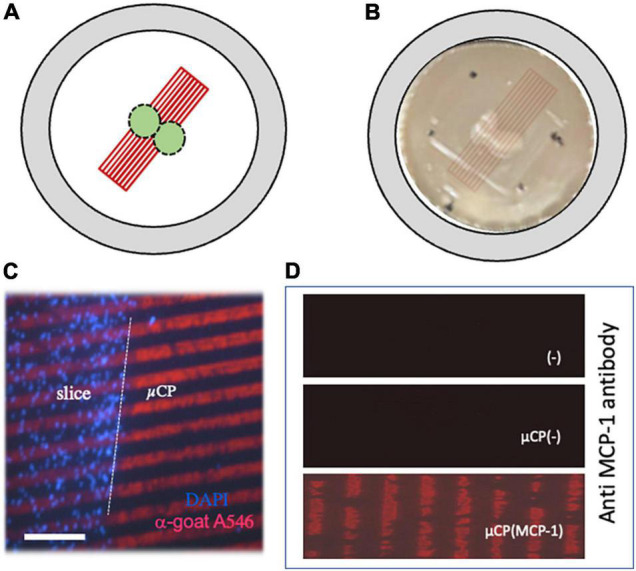
Characterization of microcontact printing. Panel **(A)** shows a scheme, how two organotypic cortex “chopper” brain slices (300 μm) were connected to microcontact prints (μCP). Panel **(B)** shows the two brain slices on a semipermeable membrane and how the μCP are coupled (artificial overlay, which is not visible by eye). The dots mark the orientation to link the slices. Panel **(C)** shows a merged picture where brain slices were cultured for 3 weeks, then fixed and stained for nuclear DAPI (blue fluorescent). The slices were connected to a loading control using red fluorescent (anti-goat Alexa-546) μCP lanes. The white dotted line in panel **(C)** indicates the border of the brain slice. Immunostainings against monocyte chemoattractant-protein-1 (MCP-1) shows that MCP-1 was printed onto the membranes [Panel **(D)**, lower panel], compared to negative controls without μCP [Panel **(D)**, upper panel] and μCP with collagen only [Panel **(D)**, middle panel]. Scale bar in panel **(C)** = 500 μm **(B)**, 190 μm **(C)**, 110 μm **(D)**.

Organotypic chopper brain slices were prepared as reported in detail in our lab (Steiner and Humpel, 2021). Briefly, postnatal day 8–10 C57BL/6 wildtype mouse pups were rapidly decapitated and the brains were dissected under sterile conditions. The parietal cortex was dissected and 300 μm slices were chopped on a McILWAIN Tissue chopper. Brain slices were carefully transferred to a Isopore™ 0.4 μm pore PC membrane (HTTP02500, Merck Millipore, Darmstadt, Germany) with microcontact printed lanes (three different groups A-B-C, see below; Figure 1A). These extra membranes were transferred onto semipermeable 0.4 μm pore cell culture inserts (PICM03050, Merck Millipore, Darmstadt, Germany) which were placed into 6-well plates (Greiner Bio-One, Vienna, Austria) (see Figure 1B). Each well contained 1 ml of sterile-filtered culture medium [50% MEM/HEPES (Gibco, Vienna, Austria), 10% heat inactivated horse serum (Gibco/Lifetech, Vienna, Austria), 25% Hanks’ solution (Gibco, Vienna, Austria), 2 mM NaHCO_3_ (Merck, Austria), 6.5 mg/ml glucose (Merck, Germany), 2 mM glutamine (Merck, Germany), at pH 7.2]. Brain slices were cultured at 37°C and 5% CO_2_ for 3 weeks and culture medium was changed once a week. In some experiments slices were exposed to 1.0 μm fluorescent Microbeads (1:200; FluoSpheres, Life Technology, F8815) in the medium for 3 h.

All experiments conformed to Austrian guidelines on the ethical use of animals and were in line with the 3Rs rule as all efforts were made to reduce the number of animals. In fact, from 1 mouse pup we can generate 50–100 brain slices dependent on the area and purpose. All animal experiments were defined as “Organentnahme” according to Austrian laws. In total we sacrificed 18 mice and the experiment has been repeated three times.

For this study 3 μCP groups were prepared: membranes without μCP lanes (**Group A)**, membranes with empty collagen μCP lanes (**Group B)**, and membranes with μCP loaded with MCP-1 (**Group C).** The experimental set up was the following: after an initial incubation of 4 days under normal conditions, the slices were treated for 3 days and then incubated for further 2 weeks (in total 3 weeks in culture). On the 3 μCP groups (groups A-B-C), four different treatments were performed: **treatment 1** served as a control without any stimuli; **treatment 2** was done to maximally stimulate microglia by addition of 1 μg/ml lipopolysaccharide (LPS) for 3 days; **treatment 3** simulated ischemia with oxygen-glucose deprivation (OGD); and **treatment 4** simulated ischemia with acidosis at a pH of 6.5 for 3 days. OGD was performed as described by us (Serbinek et al., 2010). Briefly, slices were transferred to medium with low glucose (6.15 mM) and the plates were put in a Modular Incubator Hypoxia Chamber (MIC-101, Billups-Rothenberg, Inc., Del Mar, CA, United States) connected to a flow meter. The chamber was sealed and a mixture of 95% N_2_/5% CO_2_ was flushed at a flow rate of 25 L/min for 4 min. The in- and outports of the chamber were closed, and the closed air tight system was placed at 37°C in an incubator for 3 days (the flush was repeated on days 2 and 3). Measurements by the manufacturer indicate that the pO_2_ reached a nadir of 35 mm Hg after 6 h. At the end of the experiment the outlet port was slowly opened, the chamber opened, and the culture wells taken and incubated under normal conditions for additional 2 weeks. After the culture period all slices were fixed for 3 h at 4°C in 4% paraformaldehyde and stored at 4°C in 10 mM phosphate buffered saline (PBS) until use.

### Collagen-Based Microcontact Prints

Collagen hydrogels were prepared as described in detail by us (Steiner and Humpel, 2021). As a crosslinker 4S-Star-polyethylineglycole succinimidyl succinate (4S-StarPEG; JKA7006-1G, Sigma) was used. Two mg/ml sterile bovine collagen solution type I (804592-20ML, Sigma) was linked with 0.4 mM 4S-StarPEG in PBS at pH 7.4. The Collagen-PEG solution was loaded with or without recombinant mouse MCP-1/CCL2 (MedChemExpress, CatNr. HY-P7764) in a final concentration of 10 ng/μl. As an additional loading control red fluorescent Alexa-546 anti-goat antibodies (final concentration: 20 ng/μl) was used. In a supplemental experiment human beta-amyloid (42) and P301S aggregated tau were loaded. The equal volume of PBS was added for generating control collagen hydrogel microcontact prints. During the handling, all components were kept on ice to prevent premature gel formation. Approx. 100 μl of collagen hydrogel ink solution (=̂1 μg MCP-1) were immediately applied onto the μCP stamp (see below).

The μ*CP* was performed similar as reported from our lab (Steiner and Humpel, 2021). μCP uses an elastomer stamp which adsorbs the “ink” solution and transfers it to a surface with a very high resolution. Approx. 50 μl of the liquid collagen hydrogel ink solution (loaded with PBS or MCP-1 or as a loading control anti-goat Alexa-546 antibodies) were applied directly onto the micropatterned stamp. To distribute the ink solution equally, a cover slip was placed on top. After 15 min of incubation at 37°C, the cover slip was removed and used to carefully strike off the remaining ink solution, once with and once against the lanes of the pattern. Excess solution on the borders of the pattern was removed by filter paper without touching the printing surface or left to air dry for a minute. As soon as the stamp was completely dry, the ink solution was transferred to the semipermeable membrane by pressing-on with 18 grams for 60 min at room temperature. The position of the stamp was marked with a small dot of permanent marker for later arrangement of the slices. Then, the weight and the stamp were carefully removed from one corner. Membranes were sterilized under UV light for 20 min, equilibrated with slice medium and placed on inserts before arranging the brain slices.

In order to proof that MCP-1 was μCP onto the membranes, the lanes were stained by immunostainings. Briefly, membranes were fixed for 30 min with 4% paraformaldehyde directly after the μCP, shortly washed in PBS and then incubated with an anti-MCP-1 antibody (Proteintech, nr. 26161-1-AP, 1:500) overnight, washed and detected using an anti-rabbit Alexa-546 antibody.

### Immunohistochemistry for Microglia

Immunohistochemistry was performed as previously described under free-floating conditions (Steiner and Humpel, 2021). This method allows that the antibody can penetrate from both sides during incubation enhancing the sensitivity of the staining. First, the fixed brain slices were incubated in 0.1% Triton-PBS (T-PBS) for 30 min at room temperature with soft shaking. After incubation, the brain slices were washed 3 × 3 min with PBS and subsequently blocked in 20% horse serum/0.2% bovine serum albumin (BSA)/T-PBS for 30 min while shaking. Following blocking, the brain slices were incubated in 0.2% BSA/T-PBS with primary antibodies against microglial Iba-1 (Wako 019-19741; 1:500) for 2 days. After incubation the brain slices were washed 3 × 3 min with PBS and incubated with anti-rabbit green fluorescent Alexa-488 secondary antibodies (1:400 in 0.2% BSA/T-PBS) for one hour at room temperature while shaking. Brain slices were counterstained with the blue fluorescent nuclear dye DAPI (1:10,000 diluted in T-PBS) for 30 min. Brain slices were washed again with PBS before mounting them on glass slides with Mowiol. In addition, co-localization immunostainings were also performed for CD11b (abcam ab128797, 1:1000, secondary anti-rabbit Alexa-488) and macrophage-like antigen (abcam ab56297, 1:250, secondary anti-rat Alexa-546). The staining was visualized with a fluorescence microscope (Olympus BX61) and Openlab software (4.0.4).

### Data Analysis and Statistics

Quantitative analysis was performed blinded under the microscope. The number of Iba1+ migrated cells was evaluated along the slice borders at a length of 300 μm or 10 lanes on both sides and the values were averaged (1 pup = 1 n). In order to evaluate the length of the migration distance, a picture was taken under the fluorescence microscope Olympus BX61 and the pixel number of the migration was measured using OpenLab 4.0.4 software connected to a MAC computer. The number of beads was counted in a circle around the slice with a radius of 1000 μm. The sample size (*n*) gives the number of analyzed mice. All values are given as mean ± standard error of the mean (SEM). Statistical analysis was performed by one-way ANOVA with a subsequent Fisher LSD *post hoc* test, where *p* < 0.05 represents significance.

## Results

### Characterization of Microglia on Microcontact Prints Lanes

The slices were incubated for 3 weeks, attached to the membranes, flattened and became transparent. Two slices were placed in the middle of the μCP lanes, so that cells could migrate to both sides (Figure 1B). The connection of the slices to the μCP lanes is seen in Figure 1C, where slices were stained using the blue fluorescent dye DAPI and red fluorescent Alexa-546 anti-goat antibody were added as a loading control. The immunostaining shows that MCP-1 was printed onto the membranes (Figure 1D) compared to a negative control without μCP or with collagen alone (Figure 1D).

### Characterization of Iba1+ Immunostainings

**FIGURE 2:**
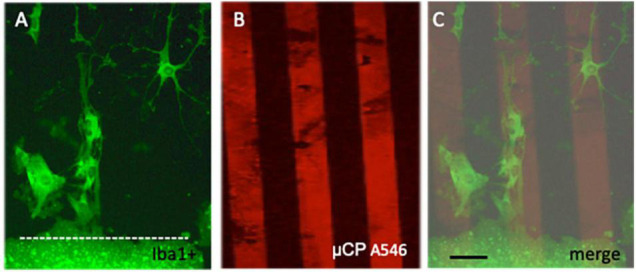
Migration of microglia along microcontact prints (μCP). Cortex brain slices were cultured on collagen-monocyte chemoattractant-protein-1 loaded μCP for 3 weeks and then fixed and stained for the microglial marker Iba1 (stained with Alexa-488, fluorescent green) **(A)**. As a loading control red fluorescent (Alexa-546 anti-goat) microprints were connected to the slice **(B)**. The merged picture **(C)** clearly shows that Iba1+ microglia migrated along the collagen μCP lanes. The slice is shown at the bottom and a white dotted line **(A,C)** indicates the border. Note several ramified Iba1+ microglia migrating out of the brain slice. Scale bar in panel **(C)** = 35 μm **(A–C)**.

**FIGURE 3:**
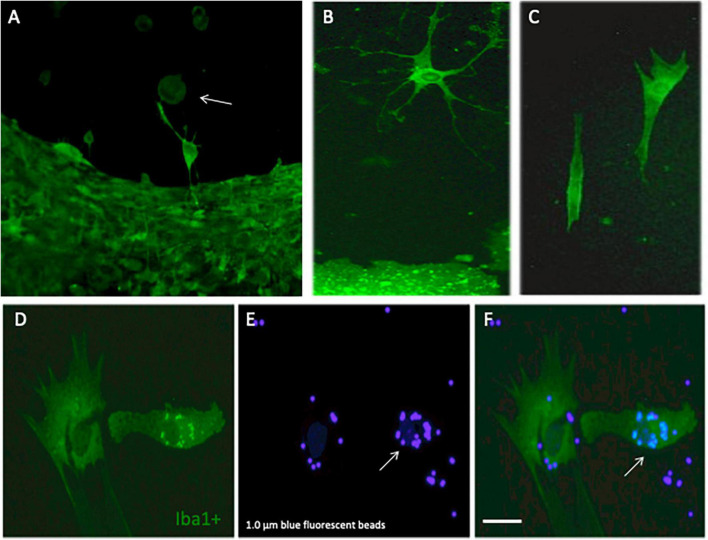
Differentiation and phagocytosis of migrated microglia. Cortex brain slices were cultured on collagen microcontact prints (μCP) loaded with monocyte-chemoattractant protein-1 (MCP-1) for 3 weeks and then fixed and stained for the microglial marker Iba1 (stained with Alexa-488, fluorescent green). Panel **(A)** shows several round ameboid microglia (arrow) migrating out of the slice and in panel **(B)** a highly ramified microglia with many processes is shown and panel **(C)** shows two elongated forms of microglia. In order to demonstrate that these microglia are capable of phagocyting beads, the slices were incubated with 1.0 μm blue fluorescent beads. Panels **(D–F)** show that migrated Iba1+ green fluorescent microglia **(D)** indeed can incorporate the blue fluorescent beads [**(E,F)**, arrow]. Scale bar in panel **(F)** = 75 μm **(A–F)**.

Microglia were immunohistochemically stained (green fluorescent Alexa-488 antibody) with the marker Iba1 and were observed to migrate out of the slices (Figure 2A). The Iba1+ cells migrated along the collagen μCP lanes (visualized as red lines in Figure 2B) which is seen in a merged picture (Figure 2C). Two different forms of migrated microglia were observed, round ameboid forms (Figure 3A, arrow) and differentiated forms. The differentiated forms occurred either as extensively arborized microglia (Figure 3B), as elongated forms with processes (Figure 3C) or as a ramified form with one large extending process (Figure 3A). In order to demonstrate that these migrated microglia have a phagocytic activity, slices were incubated with 1.0 μm blue fluorescent beads (Figure 3E). Indeed, Iba1+ microglia (Figure 3D) phagocytosed these small beads (Figure 3F).

### Effects of Microcontact Prints on Iba1+ Microglia

**FIGURE 4:**
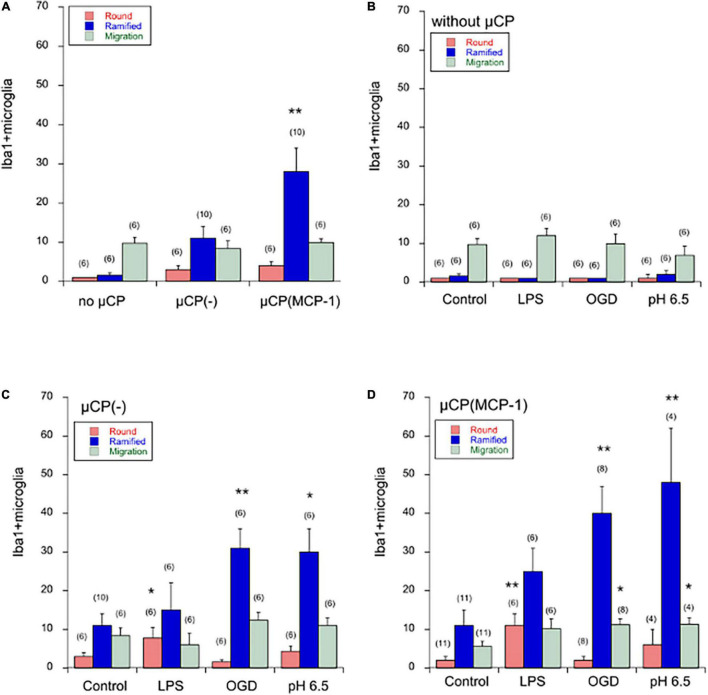
Quantification of Iba1+ microglia in the microcontact printing (μCP) assay. Brain slices were prepared, cultured for 4 days in normal slice medium and then treated for 3 days without (minus, control), or with lipopolysaccharide (LPS) or oxygen-glucose deprivation (OGD) or acidosis (pH = 6.5), and then incubated for further 2 weeks in normal slice medium. Slices were then analyzed for microglial Iba1 by immunostainings, and quantified. Panel **(A)** compares microglia cultured without any μCP or cultured on μCP without any stimulus or cultured on μCP loaded with monocyte-chemoattractant protein-1 (MCP-1). The three different treatments are compared in cultures without any μCP [Panel **(B)**], in cultures with μCP without stimulus [Panel **(C)**] and in cultures with μCP loaded with MCP-1 [Panel **(D)**]. The number of round (red bars) and ramified (blue bars) Iba1+ microglia as well as the length of migration (green bars) was counted on a length of 300 μm on both sides of the two slices and the values of four measurements averaged. For better graphical illustration of the data, the migration distance was multiplied by a factor of 10. The number of analyzed mice is given in parenthesis. Values are given as mean ± SEM cells per 300 μm or mm length. Statistical analysis was performed by ANOVA with a Fisher’s LSD *post hoc* test vs. the control group (*p ≤ 0.05; **p ≤ 0.01).

When brain slices were incubated for 3 weeks without any μCP, then very low spontaneous migration of microglia out of the brain slices was observed: 1 ± 0 round cells/300 μm, 1.6 ± 0.6 ramified cells/300 μm with a distance of 972 ± 157 μm (Figure 4A). No significant change was seen on a μCP loaded with collagen only, although there was a tendency of increased migration of ramified cells (Figure 4A). However, when slices were connected to μCP with MCP-1 then the number of ramified microglia significantly increased to 28 ± 6 cells/300 μm (Figure 4A).

### Effects on Spontaneous Migration of Microglia

Neither LPS, nor OGD, nor acidosis had any direct effect on the spontaneous migration of microglia out of the brain slice, although there was a tendency that acidosis reduced the migration length (Figure 4B).

### Effects on Microglia Along Collagen-Loaded Microcontact Prints

Treatment of cultures with LPS for 3 days significantly enhanced the number of round amoeboid microglia but not of ramified cells (Figure 4C). In contrast, OGD as well as acidosis significantly enhanced the number of migrated ramified microglia but not round cells (Figure 4C). No effect was seen on the migration distance, although there was the tendency that LPS reduced the migration length (Figure 4C).

### Effects on Microglia Along Monocyte Chemoattractant Protein-1 Loaded Microcontact Prints

Treatment of cultures with LPS again enhanced the number of round migrating cells (Figure 4D). When cultures were exposed to OGD or acidosis again the number of ramified microglia significantly increased (Figure 4D). Again, no effect was seen on the migration length of the cells (Figure 4D).

### CD11b, Macrophage-Like Antigen and Phagocytosis of Beads

**FIGURE 5:**
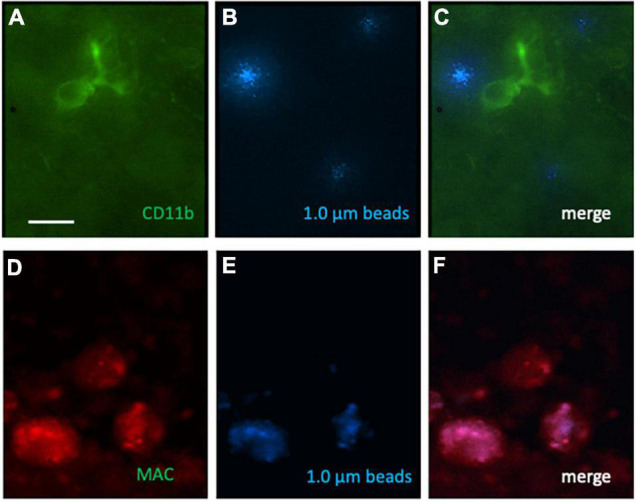
Immunostaining for CD11b, macrophage-like antigen (MAC) and phagocytosed 1.0 μm blue fluorescent beads on microcontact prints loaded with MCP-1. Ramified CD11b [green Alexa-488; **(A)**] and activated phagocytic microglia (red, Alexa-546, **D**) were found. Blue fluorescent beads **(B,E)** were phagocytosed by activated phagocytic microglia **(F)** but not ramified microglia **(C)**. Scale bar in panel **(A)** = 35 μm **(A–F)**.

In addition to Iba1, we also performed immunostainings for CD11b and macrophage-like antigen. Several ramified CD11b+ microglia (Figure 5A) and activated phagocytic microglia (Figure 5D) were seen in the migration zone. Several phagocytosed blue beads were found (Figures 5B,E), which co-localized with activated phagocytic microglia (Figure 5F) but not ramified microglia (Figure 5C). In average 8.7 ± 3.4 (*n* = 6) CD11b + microglia and 5.5 ± 2 (*n* = 6) activated phagocytic microglia per 300 μm field were counted in controls, which was not different to OGD treatments.

### Ramified Microglia on Microcontact Prints Loaded With Beta-Amyloid and Tau

**FIGURE 6:**
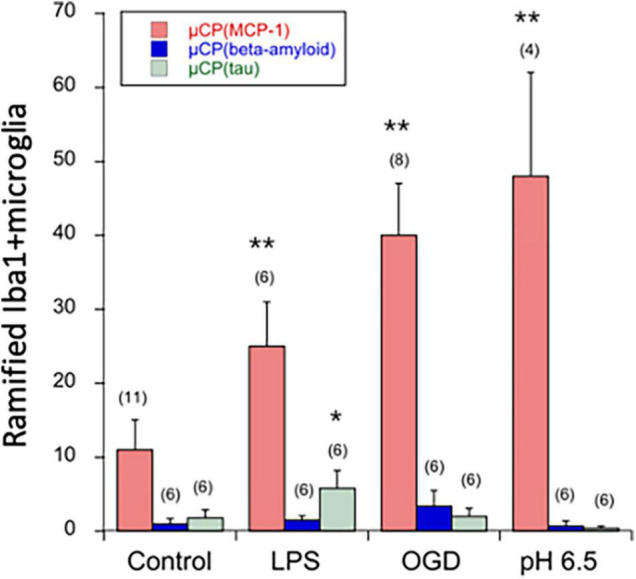
Quantification of ramified Iba1+ microglia in the microcontact printing (μCP) assay loaded with MCP-1 (red bars), beta-amyloid (blue bars), or tau (green bars). Brain slices were prepared, cultured for 4 days in normal slice medium and then treated for 3 days without (minus, control), or with lipopolysaccharide (LPS) or oxygen-glucose deprivation (OGD) or acidosis (pH = 6.5), and then incubated for further 2 weeks in normal slice medium. Slices were then analyzed for microglial Iba1 by immunostainings, and quantified on a length of 300 μm on both sides of the two slices and the values of four measurements averaged. For better graphical illustration of the data, the migration distance was multiplied by a factor of 10. The number of analyzed mice is given in parenthesis. Values are given as mean ± SEM cells per 300 μm. Statistical analysis was performed by ANOVA with a Fisher’s LSD *post hoc* test vs. the control group (*p ≤ 0.05; **p ≤ 0.01).

The number of migrated ramified Iba1+ microglia was markedly lower on μCP loaded with beta-amyloid or tau compared to MCP-1 (Figure 6). Interestingly, LPS significantly enhanced the number of migrated Iba1+ microglia on tau but not beta-amyloid μCP (Figure 6). No effects were seen with OGD or acidosis (Figure 6).

## Discussion

In the present study we show for the first time that microglia migrate along μCP lanes of collagen loaded with MCP-1 or without. Ischemic events simulated by OGD and acidosis activated the ramification of microglia, while the most potent inflammatory stimulus LPS activated round ameboid microglia.

### Organotypic Brain Slices as a Tool to Study Microglia

Organotypic brain slice cultures represent a physiologically relevant three-dimensional *ex vivo* model (see our review, Humpel, 2015, 2018). As the main architecture of the cells is preserved, it allows *in vitro* investigation of cellular and molecular processes in the brain areas of interest. Gähwiler’s group was the first who succeeded in culturing organotypic brain slices by means of the roller-tube technique (Humpel, 2015). This technique was modified, maintaining organotypic brain slices in culture on semipermeable membranes (Humpel, 2015). In our lab we use the “chopper” technique, where small 300 μm pieces are taken from the parietal cortex. This technique allows to collect 50–100 slices per mouse pup. Further, we can place two slices together onto one μCP membrane. Organotypic brain slice cultures are prepared from postnatal day 8–10 brains as the tissue and cell survival is high in this timeframe. The older the donor animals (>14 days postnatal), the more tissue and cell death occurs in culture. Brains of younger donor animals exhibit a looser texture and morphology, as they are more immature. Slices were stained after 3 weeks *in vitro* since after this period of time slices are completely flattened from 300 to approx. 100 μm. The grade of flattening is not only an important parameter of macroscopic cell survival, but also increases the quality of immunostaining and microscopic analysis. The thicker the slices, the less antibodies are able to diffuse deep into the slice and images may appear more blurry. However, it is an advantage that we performed all immunostainings free-floating, as antibodies are then able to penetrate the slice from both sides improving image quality. In the present study we used slices derived from the parietal cortex, where we previously have shown that microglia survive and get activated due to GM-CSF (Schermer and Humpel, 2002; Zassler et al., 2003). Microglia were stained using the well-established microglial marker Iba1+ (Shapiro et al., 2009) and round as well as ramified microglia could be identified.

### Slice, Microcontact Printing, and Collagen Loads

In the present study we coupled an organotypic brain slice with μCP lanes and studied the effects on microglia. In the first step, we observed the migration of microglia without any μCP and found that only a few round and ramified microglia (approx. 2 per field) were seen outside the slice. However, when μCP loaded with collagen only were coupled to the slice, then a higher number of ramified microglia was seen (approx. 11 per field). This indicates that round cells migrated out along the collagen μCP and differentiated there within 3 weeks. When slices were coupled to μCP and loaded with MCP-1 in collagen, then the number of ramified microglia was even higher (approx. 28 per field). This clearly indicates that MCP-1 stimulated the migration of microglia along the μCP lanes. In general, the effects of LPS, OGD, and acidosis were not evident in slices without any μCP lanes, but definitely more pronounced when collagen μCP lanes were connected. This clearly shows that collagen alone is a potent stimulus for the microglia to get activated. However, when the slices were connected to μCP with MCP-1, the number of ramified microglia significantly increased. We cannot exclude that some microglia underwent cell death due to the very strong (3 days) stimuli with LPS, OGD or acidosis. Further experiments with low stimuli (e.g., 1 instead of 3 days) and shorter culture time (2 instead of 3 weeks) could show more significant effects.

### The Effect of Monocyte Chemoattractant Protein-1 on Microglial Migration

Microglia secrete numerous biologically active molecules including cytokines and growth factors, which are involved not only in the regulation of immune responses but also in the process of tissue repair (Yadav et al., 2010; Schuette-Nuetgen et al., 2012). MCP-1 (or CCL2) belongs to a large family of structurally related small cytokines and regulates the migration of leukocytes after inflammation and immune response (Yadav et al., 2010; Schuette-Nuetgen et al., 2012; Yao and Tsirka, 2014). MCP-1 has been reported to be induced in the brain after cerebral ischemia and is possibly involved in immune processes causing neuronal death and activating microglia (Sakurai-Yamashita et al., 2006). MCP-1 is produced by many cell types known to be important for immune responses, but amoeboid microglial cells were found to be the major source of MCP-1. Upon hypoxic insult, amoeboid microglial cells even increase the MCP-1 production and recruit more microglia. It is well established that MCP-1 regulates migration and infiltration of microglia, monocytes, but also lymphocytes to the inflammatory sites in the brain. Indeed, MCP-1 signaling mediates microglial activation and neuroinflammation that demonstrably contributes to neurodegeneration. In the present study, we have used MCP-1 to stimulate migration along the μCP lanes. Here we show that microglia indeed showed a higher potency to migrate along the MCP-1 μCP and displayed a higher ramification.

### The Effect of Lipopolysaccharide on Microglial Migration

The endotoxin LPS is a well-established molecule to induce chronic inflammation *in vitro* or *in vivo* (Qin et al., 2007). LPS is a component of the cell wall of gram-negative bacteria and stimulates the production of different pro-inflammatory cytokines from activated glial cells (Martinez and Gordon, 2014; Orihuela et al., 2016). In the present study we have used LPS to maximally stimulate microglia. Our data show that LPS activated microglia to migrate along the collagen μCP. This effect was also seen on μCP loaded with MCP-1 but was not markedly potentiated. Interestingly, the LPS effects were not accompanied by ramification of the microglia. In order to investigate if the two Alzheimer markers beta-amyloid and tau may affect Iba1+ microglia, we loaded both substrates into the μCP lanes. Our data show, that very few microglia migrated compared to the MCP-1 control. However, we show that Iba1 + microglia migrated and differentiated on tau μCP when stimulated with LPS. This is an interesting preliminary observation and need further investigation for the mechanisms. No effects were seen for OGD or acidosis or beta-amyloid.

### The Effect of Acidosis on Microglial Migration

It is now widely accepted that acidosis is an important component of the pathological event which leads to ischemic brain damage. Acidosis is a result of either an increase in tissue CO_2_ or an accumulation of acids produced by dysfunctional metabolism. Severe hypercapnia (arterial CO_2_ around 300 mm Hg) may cause a fall in tissue pH to around 6.5 without any morphological evidence of irreversible cell damage. In severe ischemia and tissue hypoxia, anaerobic glycolysis leads to accumulation of acids, example lactate, causing a decrease in pH to around 6.0 with strong signs of irreversible damage. This cellular damage seems to be mediated by free radicals but not by a perturbation of cell calcium metabolism. In the present study we used the model of moderate acidosis (pH 6.5) in order to activate microglia for a period of 3 days. Our data show that acidosis caused an activation of microglia for ramification, which was seen in collagen μCP without load and more pronounced when loaded with MCP-1. This goes in line, with an ischemic property of low pH.

### The Effect of Oxygen-Glucose Deprivation on Microglial Migration

The OGD model in slice cultures has been widely used to study “ischemia-like” conditions (Girard et al., 2013; Ziemka-Nałęcz et al., 2013) and is a combined model of loss of oxygen and glucose. Our present model uses a mild and more prolonged OGD set up to simulate ischemic events and we have described this model in brain slices in a previous study (Serbinek et al., 2010). In the present study we applied OGD for 3 days, as we have shown that prolonged OGD is problematic, because the pH markedly decreases due to lactate production and damages the slices. Our data show that OGD activated microglia to migrate out of the slices and to get ramified. Again, this effect was seen in collagen only μCP and more enhanced in the MCP-1 loaded μCP. The effect was nearly identical to the one seen after acidosis. In addition to Iba1, we also stained for CD11b and MAC, and our data confirm that ramified cells stained for CD11b and activated phagocytic microglia. We also show that 1.0 μm beads were selectively phagocytosed by activated phagocytic microglia but not ramified cells. The OGD treatment did no markedly affect the differentiation to CD11b ramified microglia or activated phagocytic microglia. Possibly longer incubation times may be necessary.

### Limits of the Study and Outlook

This study has some limits: (1) The number of animals per groups (*n* = 6) was sufficient to reach significance, but the heterogeneity in slice preparations caused some fallout and elimination of some slices. (2) In the present study we used Iba1 and CD11b and MAC antigen to stain microglia. The use of markers to differentiate between pro-inflammatory or anti-inflammatory M1/M2 markers could give additional better insights into mechanistic processes. (3) The aim of the present study was to show proof-of-principle that slices coupled to μCP are a potent model to study microglial migration. Further, the use of the biomaterial collagen was superior to print the lanes. Thus, we did not have in focus to explore any mechanistic effects on the stimulation and migration or differentiation of microglia. (4) As mentioned, we have used a maximal stimulation over 3 days with LPS, OGD and acidosis. A milder stimulation or shorter or longer culture time may have more pronounced effects. (5) We have not included any reductionist approach, such as example to block the effects of migration with example anti-MCP-1 antibodies or siRNA technology. (6) In the present study we focused on the migration along the μCP lanes, and we were not interested what happens within the brain slices. All three stimuli will definitely affect the microglia within the slice and activate them to migrate out. (7) It was very interesting to note that we did not see any effects on the migration length. In most cases all round migration microglia migrated at least 1000 μm and we did not differentiate if 1 or 10 cells were found. In any case we suggest that first round microglia migrate and second differentiate. (8) In additional studies it would be interesting to test a gradient of the loaded cytokine and to perform a time-dependent experiment (e.g., 2-4-7 days).

While this study has some limits, it also highlights the potency of such a model. First, using this technique we can print nearly any protein or peptide of interest on the lanes and can screen any molecule on the migration of microglia. Second, we can also investigate other functions in the slices, such as nerve fiber growth or vessel growth. Third, the slice model is an *in vitro* model which contributes to the 3Rs of animal experimentation and reduces animal numbers, as we can prepare up to 100 slices per mouse. Forth, in this model, we cannot only study ischemia-like events, but also many other activators, such as toxins or receptor agonists. And finally, we claim that biomaterials, such as collagen are very potent to print proteins in lanes, but this also suggests very likely, that other biocompatible biomaterials (such as alginate) but also other materials (such as nanoparticles) may be useful in printing lanes on a membrane.

In summary, we show for the first time that microglia migrate out from brain slices along microcontact printed lanes. This effect was not seen without any μCP lanes, but was stimulated with collagen alone, while MCP-1 stimulated the outgrowth. Ischemic events were simulated by OGD and acidosis, both displaying the same effect, namely, the induction of microglial ramification. In contrast, LPS had another effect and only stimulated migration of round ameboid microglia. In conclusion, our brain slice model coupled to collagen-loaded μCP is very potent to explore the migratory capacity of microglia in a simple *ex vivo* model.

## Data Availability Statement

The raw data supporting the conclusions of this article will be made available by the authors, without undue reservation.

## Ethics Statement

Ethical review and approval was not required for the animal study because all experiments conformed to Austrian guidelines on the ethical use of animals and were in line with the 3Rs rule as all efforts were made to reduce the number of animals. All animal experiments were defined as “Organentnahme” according to Austrian laws and are not animal experiments.

## Author Contributions

KS performed all experiments. CH designed and analyzed the data. Both authors wrote the manuscript, contributed to the article, and approved the submitted version.

## Conflict of Interest

The authors declare that the research was conducted in the absence of any commercial or financial relationships that could be construed as a potential conflict of interest.

## Publisher’s Note

All claims expressed in this article are solely those of the authors and do not necessarily represent those of their affiliated organizations, or those of the publisher, the editors and the reviewers. Any product that may be evaluated in this article, or claim that may be made by its manufacturer, is not guaranteed or endorsed by the publisher.
